# Sociodemographic and behavioral factors related to obesity among adults in the Republic of Palau based on the WHO STEPwise approach to NCD risk factor surveillance 2011–2013: A cross-sectional study

**DOI:** 10.1265/ehpm.22-00309

**Published:** 2023-06-28

**Authors:** Miyuki Hasegawa, Kaori Honjo, Chifa Chiang, Takashi Mita, Berry Moon Watson, Edolem Ikerdeu, Sherilynn Madraisau, Hiroshi Yatsuya, Atsuko Aoyama, Hiroyasu Iso

**Affiliations:** 1 Department of Social Medicine, Osaka University School of Medicine, 2-2 Yamadaoka, Suita, Osaka 565-0871, Japan; 2 Social and Behavioral Science, Faculty of Medicine, Osaka Medical and Pharmaceutical University, 2-7 Daigakumachi, Takatsuki, Osaka 569-8686, Japan; 3 Department of Public Health and Health Systems, Nagoya University School of Medicine, 65 Tsurumai-cho, Showa-ku, Nagoya 466-8550, Japan; 4 Faculty of International Relations, Kyoto Sangyo University, Motoyama, Kamigamo, Kita-ku, Kyoto 603-8555, Japan; 5 Ministry of Health, Republic of Palau, One Hospital Road, Koror 96940, Republic of Palau; 6 Institute of Health and Nutrition, Nagoya University of Arts and Sciences, 57 Takenoyama, Iwasaki, Nissin, Aichi 470-0196, Japan; 7 Institute for Global Health Policy Research, National Center for Global Health and Medicine, 1-21-1 Toyama, Shinjuku-ku, Tokyo 162-8655, Japan

**Keywords:** Body mass index, Obesity, Noncommunicable disease, Micronesian country, Palau, WHO STEPwise approach to NCD risk factor surveillance

## Abstract

**Background:**

Pacific islanders face drastic increase of obesity-related noncommunicable disease (NCD) due to lifestyle shifts of unhealthy diets and physical inactivity. To date, however, obesity related factors have not been well elucidated in Republic of Palau. This study aimed to investigate sociodemographic and behavioral factors related to obesity using the national level data in Palau.

**Methods:**

This is a cross-sectional, population-based study analyzing random sampling data of 2133 adults aged 25–64 years (of 20 thousand national population) from the WHO STEPwise approach to NCD risk factor surveillance (STEPS) implemented between 2011 and 2013. Sociodemographic and behavioral factors were obtained by the STEPS standardized questionnaire for NCD risk factors plus the question on betel nut chewing because of its common behavior in Micronesian countries. Logistic regression analysis was performed to estimate multivariable odds ratio (OR) of general obesity (body mass index ≥30.0 kg/m^2^) and central obesity (waist circumference ≥90 cm in men and ≥80 cm in women).

**Results:**

Means of body mass index, prevalence of general obesity and central obesity were higher in women (29.9 kg/m^2^, 45.5% and 85.4%) than in men (29.3 kg/m^2^, 40.4% and 67.6%). After adjusted by other potential factors, native Palauan (OR 4.4, 95% CI, 2.7–7.0 for men and 3.6, 2.3–5.6 for women), betel nut chewing (1.5, 1.1–2.1 for men and 1.6, 1.2–2.3 for women), men who work at government office (1.6, 1.2–2.1), women with higher household income (1.4, 1.0–1.8) were positively associated with general obesity, while frequent vegetable intake were inversely associated with it among women (0.71, 0.54–0.93). Similar associations were observed between the aforementioned factors and central obesity.

**Conclusions:**

Native Palauan, people with betel nut chewing behavior, government employment and higher income appeared to be associated with obesity, while frequent vegetable consumption were inversely associated with obesity. Further interventions for prevention and control of obesity are necessary through the enhancing public relation activities to understand harmful health effects on betel nuts chewing and recommending domestic production of vegetables.

## Background

Pacific islanders face serious health challenge in increase of obesity for the several past decades [1]. Among United States (US) population, Native Hawaiians/Pacific Islanders were reported to have three-times higher percentage of obesity than the overall Asian American population in 2014 [2]. Obesity is one of the key factors to enhance the risk of death from noncommunicable disease (NCD), particularly in cardiovascular disease and cancer [3]. Effective strategies for prevention and control of obesity are crucial for Pacific islanders.

The Republic of Palau is a middle-income Micronesian country with tropical marine climate consisted of 386 islands in Western Pacific. It was established as an independent state after the Compact of Free Association with the US in 1994, and approximately 20 thousand national population lives making livelihood mainly by tourism industry. The prevalence of obesity in Palau was reported to be one of the highest in the world [4], and has been increasing over a last decade [5]. Even among the Pacific island countries, according to the WHO country health information profile in 2010, Palau was the top five country of highest age-standardized mortality rates of cardiovascular disease and diabetes in the general population aged over 25 years [6]. Therefore, in Palau, the government declared a state of health emergency to tackle NCD, then conducted the first national NCD survey, i.e., the World Health Organization (WHO) STEPwise approach to NCD risk factor surveillance (STEPS) 2011–2013 [7]. Previous studies analyzing this STEPS data suggested positive associations between obesity and NCDs [8–11].

In addition to genetics, factors for excess weight gain included eating and physical activity, insufficient sleep, and socioeconomic variables [12, 13]. In the Pacific islands, unhealth dietary habits, especially high consumption of sugar sweetened beverages has been recognized as an important factor for obesity epidemic [1]. Obesity has been investigated and documented as a risk factor of NCDs, including Young Risk Behavior Survey and Youth Tobacco Survey by US Centers for Disease Control and Prevention in Palau [14]. However, to date, no studies have investigated obesity-related factors comprehensively not only in Palau but in Micronesian countries. Nationwide and country-specific data can be of use to capture the population characteristics and lifestyle factors related to obesity for the NCD early prevention.

The aim of this study, hence, was to investigate sociodemographic and behavioral factors related to obesity in Palau using the population-based data of men and women aged 25–64 years.

## Methods

### Data collection

The WHO STEPS is global standardized method for NCD risk factor surveillance composed three instruments: questionnaire of behavioral risk factors on NCDs (Step 1); anthropometric measurements and resting blood pressures (Step 2); and biochemical blood tests (Step 3) [15]. A STEPS was conducted in Palau from September 2011 to June 2013 by the Ministry of Health (MOH) with financial backing from WHO. The question on a chewing behavior of betel nut, which consists of betel leaf, an areca nut, slaked lime and often added tobacco, were added because betel nut chewing is a common behavior in Micronesian countries. Previous researches suggested that betel nut chewing behavior has various harmful health effect increased with risks of cardiovascular disease and oral disease, and adverse reproductive outcomes [16–18]. Based on the 2009 Household Survey, one resident aged 25 to 64 years each from 2807 households (of the total 3976 households nationwide) were extracted by two-stage cluster random sampling covering the entire 16 states of Palau. A total of 2226 individuals participated and then used for the present analysis, and 2212 individuals of them were completed all of three steps instruments (the response rate was 79%). The details of the survey are described elsewhere [7]. The trained MOH staffs visited participants residences for face to face interviews and physical measurements. Anthropometric measurements were collected once in light indoor clothing without shoes by general portable devices. Data analysis was a part of joint research between the MOH of Palau and a Japanese research team of Osaka University and Nagoya University. Participant identification was anonymized and unlinkable for privacy policy.

### Measurements

Waist circumference (WC) was measured at the midpoint between lowest rib and iliac crest. Body mass index (BMI) was calculated as weight in kilograms divided by height in meters squared. General obesity was defined as BMI ≥30.0 kg/m^2^ in accordance with the WHO classification [19]. In this study, cut-offs for central obesity were determined as WC ≥90 cm for men and ≥80 cm for women in line with the International Diabetes Federation consensus for Asians although the appropriate WC cut-off values has not been identified for Pacific islanders [20].

### Study population

Of 2226 participants, we excluded those with missed the data of sex, age, ethnicity and resident state (n = 17), and those with missed and incorrectly inputted data of height, weight, WC (n = 73), and pregnant women (n = 3). Finally, the data of 2133 participants (1016 men and 1117 women) were used for the analysis.

### Sociodemographic and behavioral factors

The sociodemographic and behavioral factors were chosen to estimate the association with obesity by using as much as available in the STEPS questionnaire. Those factors were dichotomized to carry out logistic regression analysis. Sociodemographic factors were as follows: ethnicity (native Palauan vs. non-Palauan); residence (urban: residing in Koror/Airai states where are the most populated in Palau vs. rural: residing in other states); education attainment (higher education: equal to or higher than high school graduation vs. lower education); employment type (government employment vs. other type employment/unpaid); marital status (currently married/cohabitating vs. unmarried); household income (higher income: equal to or more than 8400 U.S. dollars per year, which was median income of total study population vs. lower income). Behavioral factors were as follows: current smoking (yes vs. no); current betel nut chewing (yes vs. no); frequency of alcohol intake (frequent: five times or more per week for the past 12 months vs. less). Frequent vegetable/fruit intake were based on the question how many days per week of intake (frequent: seven days per week vs. less). Frequent eating-out were also defined based on frequency per week (within the range of 0 to 21 times, frequent: five or more times per week vs. less). The data of occupation, travel, and recreation/sport/leisure-related physical activities were collected in the STEPS but could not be used for the present analysis because the answers were considered to be unreliable due to inappropriate methods of interview with poor interviewer’s training.

### Statistical analysis

The results of continuous variables were presented as mean value with standard deviation and categorical variables were presented as proportion. The analysis of covariance was performed to compare age adjusted proportions of all factors as a mean value with standard error or as proportion according to general obesity (yes vs. no) and central obesity (yes vs. no) by sex. Logistic regression analysis was used to estimate sex-specific multivariate odds ratio (OR) and 95% confidence interval (CI) of general and central obesity. Two sided *p* values <0.05 were regarded as statistical significance. All statistical analyses were conducted using SAS ver.9.4 (SAS Institute Inc., Cary, NC, United States).

## Results

Table 1 shows sex-specific characteristics of the study populations. The mean BMI, prevalence of general obesity and central obesity were higher in women (29.9 kg/m^2^, 45.5% and 85.4%, respectively) than in men (29.3 kg/m^2^, 40.4% and 67.6%, respectively). Approximately 70% of men and women were native Palauan. More than half had a betel nut chewing behavior, especially higher at 60.9% of women. Women had higher frequencies of vegetable and fruit intake than men. Compared with the lower educated group, the higher educated group had the larger proportions of both government employment in women (9% vs. 33%) and higher income for both sexes (33% vs. 50% for men and 23% vs. 52% for women, respectively).

**Table 1:** Sex-specific characteristics of the study population

	**Men**	**Women**	***p* value**
No. of subjects	1016	1117	-
Age, years (SD)	45.2 (10.4)	45.6 (10.2)	0.303
Height, cm (SD)	166.8 (18.8)	156.6 (17.3)	<.001
Weight, kg (SD)	81.9 (7.4)	73.5 (6.8)	<.001
BMI, kg/m^2^ (SD)	29.3 (6.1)	29.9 (6.6)	0.045
Waist circumference, cm (SD)	97.2 (14.4)	95.8 (15.3)	0.038
Hip circumference, cm (SD)	101.6 (12.1)	104.9 (14.2)	<.001
General obesity^a)^, n (%)	410 (40.4%)	508 (45.5%)	0.017
Central obesity^b)^, n (%)	687 (67.6%)	954 (85.4%)	<.001
Palauan, n (%)	730 (71.9%)	857 (76.7%)	0.010
Urban residence^c)^, n (%)	627 (61.7%)	714 (63.9%)	0.292
Higher education^d)^, n (%)	834 (82.9%)	950 (85.7%)	0.081
Government employment, n (%)	408 (40.2%)	332 (29.7%)	<.001
Higher household income^e)^, n (%)	474 (46.7%)	539 (48.3%)	0.460
Currently married, n (%)	663 (65.6%)	689 (62.4%)	0.116
Ever smoking, n (%)	575 (56.6%)	429 (38.4%)	<.001
Current smoking, n (%)	251 (24.7%)	107 (9.6%)	<.001
Current betel nut chewing, n (%)	553 (54.4%)	680 (60.9%)	0.003
Current alcohol drinking^f)^, n (%)	94 (9.3%)	31 (2.8%)	<.001
Frequent vegetable intake^g)^, n (%)	357 (35.1%)	493 (44.1%)	<.001
Frequent fruit intake^g)^, n (%)	161 (15.8%)	234 (20.9%)	0.001
Frequent eating out^h)^, n (%)	241 (23.7%)	203 (18.2%)	0.002

BMI: body mass index, SD: standard deviation.

a) BMI ≥30.0 kg/m^2^, b) Waist circumference ≥90 cm for men or ≥80 cm for women, c) Residents in Koror and Airai states, d) Equal to or higher than high school graduation, e) Equal to or more than 8400 U.S. dollars per year, f) five times or more per week during the last 12 months, g) seven days per week, h) 5 to 21 times per week.

Table 2 shows sex-specific, age-adjusted proportions of sociodemographic and behavioral factors according to the presence or absence of general and central obesity. Both general and central obesity groups had the larger proportions of native Palauan, government employment, higher income and betel nut chewing in both sexes, and currently married in women. In contract, these groups had the smaller proportions of current smoking in men and frequent vegetable/fruit intake in women. In addition, the general obesity group had the larger proportion of frequent eating out in women. The central obesity group also had the larger proportions of higher education and currently married in men, while it had the smaller proportions of urban residence and frequent alcohol drinking in women.

**Table 2:** Age-adjusted proportions of sociodemographic and behavioral factors in general obesity or central obesity group

	**General obesity^a)^**			**Central obesity^b)^**		
	
	**No**	**Yes**	***p* value**	**No**	**Yes**	***p* value**
**MEN**						
No. of subjects	606	410	-	329	687	-
Age, years (SD)	44.8 (10.6)	45.8 (10.0)	0.148	42.5 (10.4)	46.5 (10.1)	<.001
Height, cm (SE)	166.9 (0.3)	166.7 (0.4)	0.643	164.5 (0.4)	167.9 (0.3)	<.001
Weight, kg (SE)	71.0 (0.5)	98.0 (0.7)	<.001	65.6 (0.8)	89.7 (0.6)	<.001
BMI, kg/m^2^ (SE)	25.4 (0.2)	35.1 (0.2)	<.001	24.3 (0.3)	31.8 (0.2)	<.001
Waist circumference, cm (SE)	89.8 (0.5)	108.1 (0.6)	<.001	82.7 (0.6)	104.1 (0.4)	<.001
Palauan, %	59.3	90.4	<.001	51.1	81.8	<.001
Urban residence^c)^, %	63.7	58.8	0.119	61.1	62.0	0.798
Government employment, %	29.8	55.5	<.001	24.9	47.5	<.001
Higher education^d)^, %	81.8	84.6	0.245	78.8	84.8	0.019
Higher household income^e)^, %	39.0	58.0	<.001	30.4	54.5	<.001
Currently married, %	63.7	68.5	0.101	61.3	67.7	0.042
Current smoking, %	28.4	19.2	0.001	33.4	20.6	<.001
Current betel nut chewing, %	43.9	70.0	<.001	39.6	61.5	<.001
Frequent alcohol drinking^f)^, %	9.7	8.6	0.553	9.4	9.2	0.883
Frequent vegetable intake^g)^, %	38.0	32.9	0.097	33.5	37.1	0.280
Frequent fruit intake^g)^, %	17.8	13.4	0.061	17.0	15.6	0.552
Frequent eating out^h)^, %	8.1	8.0	0.975	9.3	7.5	0.336
**WOMEN**						
No. of subjects	609	508	-	163	954	-
Age, years (SD)	45.1 (10.4)	46.3 (9.9)	0.046	41.4 (10.1)	46.4 (10.1)	<.001
Height, cm (SE)	156.7 (0.3)	156.4 (0.3)	0.510	153.8 (0.5)	157.0 (0.2)	<.001
Weight, kg (SE)	62.0 (0.5)	87.3 (0.5)	<.001	51.6 (0.5)	77.2 (0.5)	<.001
BMI, kg/m^2^ (SE)	25.2 (0.2)	35.6 (0.2)	<.001	21.8 (0.5)	31.3 (0.2)	<.001
Waist circumference, cm (SE)	86.7 (0.5)	106.8 (0.5)	<.001	73.4 (1.0)	99.7 (0.4)	<.001
Palauans, %	64.0	92.0	<.001	40.6	83.0	<.001
Urban residence^c)^, %	66.1	61.3	0.102	71.2	62.7	0.040
Government employment, %	22.4	38.5	<.001	16.3	32.0	<.001
Higher education^d)^, %	86.8	84.4	0.255	90.1	84.9	0.092
Higher household income^e)^, %	41.4	56.5	<.001	37.8	50.0	0.004
Currently married, %	59.5	65.7	0.033	51.3	64.2	0.002
Current smoking, %	9.0	10.3	0.488	9.1	9.7	0.829
Current betel nut chewing, %	48.2	76.0	<.001	24.8	67.0	<.001
Frequent alcohol drinking^f)^, %	3.1	2.4	0.474	8.0	1.9	<.001
Frequent vegetable intake^g)^, %	51.3	35.5	<.001	62.4	41.0	<.001
Frequent fruit intake^g)^, %	25.2	15.8	<.001	29.2	19.5	0.005
Frequent eating out^h)^, %	14.8	22.2	0.001	14.8	18.7	0.234

BMI: body mass index, SD: standard deviation, SE: standard error.

a) BMI ≥30.0 kg/m^2^, b) Waist circumference ≥90 cm for men or ≥80 cm for women, c) Residents in Koror and Airai states, d) Equal to or higher than high school graduation, e) Equal to or more than 8400 U.S. dollars per year, f) five times or more per week during the last 12 months, g) seven days per week, h) 5 to 21 times or more per week.

Tables 3 displays sex-specific multivariable ORs for general and central obesity according to sociodemographic and behavioral factors. Native Palauan had the highest ORs of general obesity in men (OR 4.4, 95% CI, 2.7–7.0) and women (3.6, 2.3–5.6) compared with non-Palauan. Men who worked at government office (1.6, 95% CI, 1.2–2.1) and women with higher household income (1.4, 1.0–1.8) had also the higher ORs of general obesity than men who worked at the other places and women with lower income, respectively. Among the behavioral factors, betel nut chewers had the higher ORs of general obesity in both men (1.5, 95% CI, 1.1–2.1) and women (1.6, 1.2–2.3) than non-chewers. In contract, the ORs of general obesity for women with frequent vegetable were lower (0.71, 0.54–0.93) compared with those with less intake. Similar associations were observed between the aforementioned factors and central obesity. Additional factors showing the higher ORs of central obesity were men with urban residence, higher household income, frequent vegetable intake and currently married women. In men, central obesity was associated with urban residence (OR 1.4, 95%CI 1.0–2.0), while general obesity was not (1.1, 0.81–1.4). In women, betel nut chewing was more strongly associated with central obesity (2.7, 1.6–4.7) compared to general obesity. Additional factors showing the lower ORs of central obesity were men with currently smoking and women with frequent alcohol.

**Table 3:** Multivariable odds ratios of general obesity and central obesity according to sociodemographic and behavioral factors

	**General obesity^a)^**				**Central obesity^b)^**			
	
	**No. of outcome/No. at risk**		Multivariable-adjusted*		**No. of outcome/No. at risk**		Multivariable-adjusted*	
	
	**With factor**	**Without factor**	**OR (95%CI)**	***p* value**	**With factor**	**Without factor**	**OR (95%CI)**	***p* value**
**MEN**								
Palauan	373/730	37/286	4.38 (2.73–7.03)	<.001	569/730	118/286	3.73 (2.40–5.79)	<.001
Urban residence^c)^	240/627	170/389	1.08 (0.81–1.44)	0.620	421/627	266/389	1.41 (1.02–1.95)	0.037
Government employment	228/408	182/608	1.59 (1.18–2.14)	0.002	326/408	361/608	1.45 (1.02–2.05)	0.038
Higher education^d)^	345/834	63/172	1.20 (0.82–1.75)	0.340	578/834	104/172	1.46 (0.99–2.15)	0.056
Higher household income^e)^	239/474	171/542	1.33 (0.99–1.80)	0.061	377/474	310/542	1.72 (1.24–2.40)	0.001
Currently married	283/663	126/347	1.26 (0.93–1.71)	0.135	471/663	211/347	1.31 (0.96–1.81)	0.094
Current smoking	78/251	332/765	0.77 (0.55–1.08)	0.127	139/251	548/765	0.60 (0.43–0.84)	0.003
Current betel nut chewing	287/553	123/463	1.47 (1.06–2.06)	0.022	422/553	265/463	1.21 (0.82–1.76)	0.338
Frequent alcohol drinking^f)^	35/94	371/913	0.72 (0.45–1.16)	0.177	62/94	618/913	0.78 (0.48–1.28)	0.330
Frequent vegetable intake^g)^	129/357	281/659	1.03 (0.75–1.40)	0.868	245/357	442/659	1.62 (1.16–2.26)	0.005
Frequent fruit intake^g)^	54/161	356/855	0.93 (0.62–1.41)	0.741	106/161	581/855	1.09 (0.71–1.68)	0.682
Frequent eating out^h)^	113/241	297/775	1.23 (0.88–1.71)	0.233	172/241	515/775	1.16 (0.80–1.69)	0.428
**WOMEN**								
Palauan	469/857	39/260	3.56 (2.26–5.60)	<.001	793/857	161/260	3.31 (1.93–5.68)	<.001
Urban residence^c)^	311/714	197/403	1.06 (0.81–1.39)	0.677	597/714	357/403	1.10 (0.73–1.68)	0.647
Government employment	194/332	314/785	1.30 (0.96–1.77)	0.087	302/332	652/785	0.97 (0.58–1.63)	0.911
Higher education^d)^	425/950	80/159	0.73 (0.50–1.07)	0.107	805/950	145/159	0.63 (0.33–1.21)	0.164
Higher household income^e)^	288/539	220/578	1.36 (1.03–1.81)	0.033	479/539	475/578	1.17 (0.76–1.81)	0.467
Currently married	332/689	171/416	1.25 (0.96–1.64)	0.101	609/689	335/416	1.60 (1.08–2.36)	0.019
Current smoking	52/107	456/1010	1.13 (0.73–1.75)	0.577	92/107	862/1010	1.11 (0.57–2.14)	0.761
Current betel nut chewing	386/680	122/437	1.64 (1.18–2.27)	0.003	638/680	316/437	2.73 (1.58–4.71)	<.001
Frequent alcohol drinking^f)^	12/31	496/1084	0.69 (0.31–1.51)	0.350	18/31	934/1086	0.12 (0.05–0.28)	<.001
Frequent vegetable intake^g)^	181/493	327/624	0.71 (0.54–0.93)	0.013	393/493	561/624	0.55 (0.36–0.82)	0.004
Frequent fruit intake^g)^	82/234	426/883	0.72 (0.51–1.02)	0.064	190/234	764/883	0.98 (0.61–1.56)	0.920
Frequent eating out^h)^	111/203	397/914	1.19 (0.84–1.67)	0.325	175/203	779/914	0.74 (0.44–1.26)	0.246

OR: odds ratio, 95%CI: 95% confidence interval.

*Adjusted for all other covariates.

a) BMI ≥30.0 kg/m^2^, b) Waist circumference ≥90 cm for men or ≥80 cm for women, c) Residents in Koror and Airai states, d) Equal to or higher than high school graduation, e) Equal to or more than 8400 U.S. dollars per year, f) five times or more per week during the last 12 months, g) seven days per week, h) 5 to 21 times or more per week.

Given the results that the highest ORs were shown among native Palauan, we carried out additional multivariable analyses restricted to native Palauans (not shown in Table). Among men, the higher ORs of both general and central obesity were observed for government employment (1.6, 95% CI, 1.2–2.2 and 1.5, 1.0–2.2, respectively) and higher income (1.5, 1.1–2.1 and 1.8, 1.2–2.7, respectively) compared with non-government employment and lower income, respectively. Among women, the results were similar with that in total population: higher income for general obesity (1.4, 95% CI, 1.0–1.9); betel nut chewing for central obesity (2.3, 1.3–4.1); and frequent vegetable intake for general obesity (0.69, 0.51–0.92) and for central obesity (0.47, 0.27–0.81).

## Discussion

The prevalence of obesity in Palau (40% for men and 45% for women) were slightly higher among men and lower among women when compared with the other three Micronesian STEPS data from Chuuk and Pohnpei of the Federated States of Micronesia and the Republic of the Marshall Island (31–38% and 52–63%, respectively) [21]. The mean values of the WCs (97 cm for men and 96 cm for women) in Palau were also similar to the other Micronesian countries (93–104 cm and 94–102 cm, respectively) [21]. Our result that women had a higher prevalence of obesity than men, was consistent with the findings from other Pacific island countries [6]. Besides, the prevalence of general obesity in Palau was three times higher than the global prevalence (11% and 15%, respectively) according to the 2014 WHO report [22], and the prevalence of central obesity was much higher in Palau (68% and 85%, respectively) than those from two Asian reports (China: 35% and 52%, Malay: 30% and 47%, respectively) [23, 24] using the same WC cut-offs.

In our study, native Palauan had three to five times higher prevalence of general and central obesity than non-Palauan. Pacific islanders are reported to be genetically predisposed to obesity since they acquired abilities to adapt to their poor environment of food security when originally settled in the Pacific region, thus they may have hereditary traits for elevated risk of obesity (e.g., a genotype of increased leptin levels in Micronesians) [25], and they do not have negative image of obesity in terms of body image perception [26]. More importantly, Pacific islanders have undergone lifestyle shifts toward unhealthy diets and physical inactivity since the late 1960s [27]. Prior to pre-Western contact, they used to consume low-fat and high-fibre foods (e.g., vegetables, fruits, seafood and taro), but are now heavily reliant on fatty, salty and refined high calorie foods (e.g., processed meats, white rice, snacks and sugar-sweetened beverages). Moreover, the WHO country monitoring indicated that almost half of adults were physically inactivity in Palau [5]. Most of non-Palauan were immigrant physical workers engaging in private service sectors for tourism at hotels, restaurants and marine leisure, or architectural engineer or domestic helper in urban areas, and they could have benefitted from healthy worker effects because government deports foreigners to their home countries whenever any health problems are found at annual health checkups. Nevertheless, Filipinos who comprised majority of immigrant workers had the relatively higher prevalence of general obesity (9.2% for men and 11.3% for women) in this study when compared to those in the home country according to Diabetes Philippines 2016 country profile (3.4% and 6.1%, respectively) [28]. In addition, urban residency appeared to be positively associated with central obesity in men even after adjustment for ethnicity and other potential factors, which suggests that the urbanized lifestyle lead to develop obesity in this country.

Among the sociodemographic factors, government employment and higher household income were positively associated with general and/or central obesity in both of total study population and native Palauan, especially among men. Government officers are likely to have low physical activity at their work with sedentary work or low physical job demand. And people with higher income tend to be physically inactive in their daily lives depending on automobile transportation and immigrant workforce for housekeeping. Regarding marital status, most of Micronesian island countries including Palau are traditionally matrilineal societies where women are core of their clans and inherit properties through matriline line [29]. Married status was positively associated with central obesity among women in this study, which could also reflect on the more stable in household economy. Our finding was in line with a result using the data from the STEPS in the Federated States of Micronesia (n = 1638) that showed the paid employee had three times higher OR of central obesity than the unpaid employee in men [30]. Meanwhile, higher educational status was not associated with obesity in both sexes in the present study, probably because the majority of this population belonged to high school graduation, but high-achieving students have opportunities to leave Palau to enter the college or university in the other islands/countries. In Palau, economically affluent people are likely to develop obesity because of their more urbanized and sedentary lifestyle, especially among men.

The proportion of current smoking in Palau (24.7% for men and 9.6% for women) was similar with that in the global population [31], and was lower than in the other Pacific islanders [6]. Current smoking was inversely associated with central obesity among men in this study corresponded to the previous report that body weight or BMI was lower among cigarette smokers than among nonsmokers [32]. Besides, among Palauan, betel nuts chewing, sometimes added tobacco, is more popular than using manufactured tobacco products as a reasonable recreational item because of easy accessibility. Our study population had much higher proportion of daily betel-nut chewers, and betel nut chewing behavior was positively associated with obesity, especially among women. A previous study of 1070 males and females Pakistanis aged 16–75 years with the use of Asian specific WC cut-off showed that areca nut chewers had the higher ORs of central obesity (OR 1.42, 95% CI: 0.97–2.06) and areca nut plus tobacco additive chewers did so (1.65, 1.03–2.63) compared to non-chewers [33]. That study also showed that areca nut chewing was associated with metabolic syndrome, with stronger association in females than in males (4.22: 2.40–7.41 and 2.85: 1.47–5.53, respectively) [33]. A study of 1049 Taiwanese men aged ≥40 years also revealed positive associations of betel nut chewing with general (BMI ≥25.0 kg/m^2^) and central obesity (WC ≥90 cm): the ORs of general and central obesity for low betel nut users (<76 quids/day x year) vs no users were 1.78 (95%CI, 1.07–2.96) and 1.19 (0.70–2.02), respectively; and those for high betel nut users (≥76 quids/day x year) vs no users were 2.01 (1.18–3.41) and 1.80 (1.10–3.23), respectively [34]. Our results on the associations of betel nut chewing with general and central obesity were consistent with these previous study findings. Underlying physiological mechanisms for association between betel nut chewing and obesity remain unclear; however, it has been suggested that betel nut extract and arecoline which have inhibitory effects on the gamma aminobutyric acid receptor may contribute to enhance obesity due to increased appetite and diabetogenic potential on adipocytes [35, 36].

Frequent eating-out is thought to be an important indicator of urbanization. However, the proportion of frequent eating-out was low and showing no association with obesity in this study. Palauan usually takes home-cooked meal or take out ‘fast-food’ become the restaurant industry developed and targeted for foreign tourists. Likewise, the proportion of people with a frequent alcohol drinking habit was very low in this study of ages 25–64 years as well as a previous report of ages 18–24 years old in Palau [37] probably because the imported alcohol beverages were expensive. Frequent vegetable intake was inversely associated with obesity and/or central obesity among women in both of total population and native Palauan. This finding was in line with a recent systematic review of ten cohort studies from the US, Europe and Asia reporting that a majority of studies supported an inverse association between vegetable intake and risk of weight gain, overweight or obesity in general populations [38]. Another systematic review of 12 experimental and 11 longitudinal studies showed that the relationship of fruit and vegetable intake with adiposity was accompanied by reduced total energy intake and increased fibre intake [39]. Low frequencies of fruits and vegetables intake are common across Pacific island countries: More than 80% of populations with less than five daily servings per day [6]. The mean values of vegetable intake frequency in Palau were higher (4.3 and 4.8 days/week in men and women) than in other Micronesians (2.6–3.7 and 2.8–3.7 days/week, respectively) [21]. However, the survey for young adults in Palau revealed that a quarter did not consume at least one serving of fruit and vegetable per day, and only 9.2% consumed five daily servings (400 g) per day [40] recommended in the WHO guideline for optimal health benefits. In Palau, a decline in agriculture and loss of local food production has resulted in low availability of vegetables, which accelerated the high accessibility of processed and imported food [27]. The improvement of vegetables availability could be beneficial for increasing this consumption in this country.

To the best of our knowledge, this was the first to investigate sociodemographic and behavioral factors related to obesity using the national level data among Micronesian islanders. However, some limitations need to be considered when interpreting our findings. First, we did not obtain the reliable data of direct key drivers to evaluate the obesity-related factors: physical activity and nutritional intake. In addition to the lack of data on physical activity, the data of nutritional intake was restricted to the questionnaires on frequency of selected foods and did not include the portion and specific food type. Second, because of its cross sectional design, we provide little evidence of causality; a planning follow-up study (including the STEPS) can reveal the longitudinal relationships of sociodemographic and behavioral factors with risk of obesity. Lastly, our study applied the specific WC cut-off of central obesity for Asian because the appropriate WC cut-offs has not been identified for Pacific islanders [20]. Definition of central obesity using optimal WC and waist hip ratio cut-off is important to determine the prevalence of central obesity and NCD risk factors in Micronesians.

In conclusion, native Palauan, having betel nut chewing behavior were positively associated with obesity, as were men with government employment and women with higher household income. In contract, frequent vegetable intake was inversely associated with obesity among women. The prevalence of obesity has been increasing for the past decades in Palau. Further interventions for prevention and control of obesity are necessary through the enhancing public relation activities to understand harmful health effects on betel nuts chewing and recommending domestic production of vegetables.

## Declarations

### Availability of data and materials

Restrictions apply to the availability of the data, which was used under a contract between public sector of Palau and a Japanese research team (Osaka University and Nagoya University). Therefore, the data cannot be shared without permission by MOH of Palau.
